# ENO1 expression and Erk phosphorylation in PDAC and their effects on tumor cell apoptosis in a hypoxic microenvironment

**DOI:** 10.20892/j.issn.2095-3941.2022.0451

**Published:** 2022-12-05

**Authors:** Huizhi Sun, Jing Mo, Runfen Cheng, Fan Li, Yue Li, Yuhong Guo, Yanlei Li, Yanhui Zhang, Xiaoyu Bai, Yalei Wang, Xueyi Dong, Danfang Zhang, Jihui Hao

**Affiliations:** 1 Tianjin Medical University Cancer Institute & Hospital, National Clinical Research Center for Cancer, Key Laboratory of Cancer Prevention and Therapy, Tianjin, Tianjin’s Clinical Research Center for Cancer, Tianjin 300060, China; 2 Department of Pathology, Tianjin Medical University, Tianjin 300070, China

**Keywords:** Pancreatic cancer, hypoxia, ENO1, apoptosis, Erk phosphorylation

## Abstract

**Objective:**

Hypoxia is an important feature of pancreatic ductal adenocarcinoma (PDAC). Previously, we found that hypoxia promotes ENO1 expression and PDAC invasion. However, the underlying molecular mechanism was remains unclear.

**Methods:**

The relationship between ENO1 expression and clinicopathological characteristics was analyzed in 84 patients with PADC. The effects of CoCl_2_-induced hypoxia and ENO1 downregulation on the apoptosis, invasion, and proliferation of PDAC cells were evaluated *in vitro* and *in vivo*. Hypoxia- and ENO1-induced gene expression was analyzed by transcriptomic sequencing.

**Results:**

The prognosis of PDAC with high ENO1 expression was poor (*P* < 0.05). High ENO1 expression was closely associated with histological differentiation and tumor invasion in 84 PDAC cases (*P* < 0.05). Hypoxia increased ENO1 expression in PDAC and promoted its migration and invasion. Apoptotic cells and the apoptosis marker caspase-3 in the CoCl_2_-treated ENO1-sh group were significantly elevated (*P* < 0.05). Transcriptomic sequencing indicated that CoCl_2_-induced PDAC cells initiated MAPK signaling. Under hypoxic conditions, PDAC cells upregulated ENO1 expression, thereby accelerating ERK phosphorylation and inhibiting apoptosis (*P* < 0.05). Consistent results were also observed in a PDAC-bearing mouse hindlimb ischemia model.

**Conclusions:**

Hypoxia-induced ENO1 expression promotes ERK phosphorylation and inhibits apoptosis, thus leading to PDAC survival and invasion. These results suggest that ENO1 is a potential therapeutic target for PDAC.

## Introduction

Pancreatic cancer is a highly fatal disease with a 5-year survival rate of less than 10% worldwide^1,2^. Most patients with pancreatic cancers are already in an advanced stage by the time of diagnosis^1^. Consequently, these patients have lost the opportunity for surgical treatment. Moreover, pancreatic cancer is not sensitive to chemotherapy and radiotherapy. Therefore, the prognosis for pancreatic cancer is poor^1,2^. Elucidating the molecular mechanisms underlying the development of pancreatic cancer and finding effective treatment targets are key to prolonging the survival of patients with this disease.

Pancreatic ductal adenocarcinoma (PDAC) is the most common histological type of pancreatic cancer^1^. Because of the large numbers of fibroblasts in the cancerous collagen of PDAC, this tumor is desmoplastic (fibrotic)^3^. Moreover, its main biological behaviors include strong invasiveness and high rates of local lymph node metastasis. PDAC is resistant to chemotherapy and radiotherapy. This biological behavior of PDAC, which differs from that of other solid tumors, may be associated with the following factors. First, the pancreas is located behind the peritoneum, and its blood supply is lower than that of the liver, stomach, and other organs in the abdominal cavity. PDAC cells grow in a hypoxic microenvironment, owing to the abundant fibrous tumor stroma and few blood vessels. Second, some studies have suggested that the hypoxic condition of pancreatic cancer enhances the characteristics of cancer stem cells, promotes invasion and metastasis, and confers resistance to radiotherapy and chemotherapy^4^. The anti-apoptosis and survival abilities of pancreatic cancer cells in the hypoxic microenvironment are the main reasons for ineffective treatment. The mitochondria of PDAC cells adapt to this situation by using respiratory chain supercomplexes. The hypoxic microenvironment induces the expression of genes involved in abnormal metabolism, invasion, and metastasis in PDAC cells. Efforts to physically disrupt the hypoxic microenvironment are important treatment strategies for patients with PDAC^5,6^. Thus, many studies have focused on identifying the molecular signaling pathways involved in apoptosis under hypoxic conditions.

Previously, we used spatial transcriptomics to investigate the hypoxia-induced spatial transcriptome distribution in human PDACs engrafted into mouse ischemic hindlimbs. We found that the molecular pathways associated with the proliferation, apoptosis, and invasion of tumor cells with the strongest invasion ability, located in the marginal area of tumor tissue, are activated in a hypoxic microenvironment. Tumor cells in this subgroup express various metabolic regulatory molecules, such as α-enolase 1 (ENO1), LDHA, TPI1, and PGK1^7^.

ENO1 is a critical glycolytic enzyme involved in the pathogenesis of various cancers^8^. ENO1 catalyzes the dehydration of 2-phosphoglycerate to phosphoenolpyruvate^9^. In addition to participating in tumor cell metabolism, ENO1 binds F-actin and tubulin, and localizes to the centrosomes, thus indicating a potential role in cell cycle regulation. Furthermore, ENO1, an RNA-binding protein, binds IRP1 mRNA and subsequently regulates the death of hepatocellular carcinoma cells^10,11^. However, the relevance of ENO1, the cell fate of PDAC in hypoxic microenvironments, and the underlying mechanisms remained elusive.

In this study, we used human PDAC samples, PDAC cell lines, and animal models to investigate the mechanism underlying the anti-apoptosis effects induced by ENO1 overexpression in pancreatic cancer cells in a hypoxic microenvironment. The results suggest treatment targets to overcome the resistance of pancreatic cancer to radiotherapy and chemotherapy, and prolong the survival of patients with this disease.

## Materials and methods

### Cell culture and chemicals

The human pancreatic cancer cell lines PANC-1, SW1990, ASPC1, BXPC3, and CFPAC1 were obtained from the Type Culture Collection Committee of the Chinese Academy of Sciences. All cells were maintained in RPMI 1,640 medium, DMED, or IMDM supplemented with 10% fetal bovine serum at 37 °C in a humidified atmosphere of 5% CO_2_ and 95% air. For hypoxia induction, a hypoxia-mimetic agent, cobalt chloride (CoCl_2_), dissolved in distilled water, was added to cell cultures at the indicated final concentrations for 24 h, whereas water was added to the culture medium alone as a vehicle control. To inhibit and activate the extracellular signal-regulated protein kinase 1/2 (ERK1/2) pathway, we used the ERK inhibitor U0126 and the ERK1/2 activator senkyunolide I (SENI), which were purchased from Selleckchem (U0126: SCH772984; SENI: S3275) and dissolved in dimethyl sulfoxide. The cells were pretreated with U0126 (10 μM), SENI (5 μM), or CoCl_2_ for 24 h.

### Patient samples

This study was approved by the Ethics Committee of Tianjin Medical University Cancer Institute & Hospital (Approval No. EK2022011). All clinical investigations were conducted in accordance with the principles of the Declaration of Helsinki. Patients were informed of the aims, methods, and other details of the study. We collected samples from 84 patients with pancreatic cancer and obtained detailed pathological and clinical data. All patients underwent surgery at the Tianjin Medical University Cancer Institute & Hospital between 2011 and 2015. The median patient age was 59.0 years (range 41–72 years). All patients had ductal adenocarcinoma, and axillary node metastases were present in 13 patients. The diameter of the primary tumor was < 2 cm in 6 patients and ≥ 4 cm in 31 patients. The follow-up period began at the time of surgery and ended in June 2016.

### Clinical evaluation of the clinical importance of ENO1 expression in human PDAC

SPSS version 20.0 was used to evaluate the data. Chi-square tests were performed to assess the pathological and clinical characteristics of the low and high ENO1 expression groups. The survival of these 2 groups was evaluated through Kaplan-Meier analysis. The relationships between ENO1 expression and pathological and clinical characteristics were evaluated with correlation analysis.

The prognostic value of ENO1 and the hypoxia-associated marker gene carbonic anhydrase (CA9)^12^ was also evaluated with the KM-Plotter online database (http://kmplot.com/). Approximately 178 PDAC patient samples were divided into 2 groups according to the median values of ENO1 and CA9 expression, on the basis of gene chip analysis. The 2 patient cohorts were compared with a Kaplan-Meier survival plot, and log-rank *P*-values were calculated.

### Immunohistochemical staining

Formalin-fixed paraffin-embedded tissues were sectioned, dewaxed, and rehydrated with a graded alcohol series. Endogenous peroxidase activity was blocked with 5% goat serum at room temperature for 10 min. The sections were heated in a microwave oven in citrate buffer for 20 min. The slides were incubated overnight with primary antibodies at 4 °C. The primary antibodies used for Western blot were anti-ENO1 (ab227978, 1:1,500; Abcam, USA), anti-CA9 (ab243660, 1:1,000; Abcam), and anti-Ki-67 (ab279653, 1:1,800; Abcam, USA). They were then washed with phosphate buffered saline (PBS) and individually incubated with biotin-labeled secondary antibodies. Color was developed with diaminobenzidine. The sections were counterstained with hematoxylin and observed under a microscope (80i; Nikon, Tokyo, Japan). Staining was scored according to a previously published method. Briefly, positive tumor cells were categorized as follows: 0, undetectable; 1, weak; 2, moderate; and 3, strong. The number of positive cells per 100 tumor cells per field was visually evaluated and scored as follows: 0, < 10% positive; 1, < 25% positive; 2, < 50% positive; and 3, > 50% positive. The staining index, or the sum of the staining intensity and positive cell scores, was used to determine the results for each sample. A sample was considered positive if the staining index was > 6. The Ki-67 index was determined at 400× magnification, and the score for each sample was defined as the average of 10 fields of view.

### Lentivirus production and transduction

The lentivirus for expression of shRNA targeting ENO1 (targeting 5′-CGTACCGCTTCCTTAGAACTT-3′) was synthesized by GeneChem (Shanghai, China). SW1990 and PANC-1 cells were transduced with shRNA targeting ENO1 (shENO1) or a non-targeting control (shNTC) and incubated with 0.5 mg/mL puromycin for 4 weeks.

### Western blot analysis

The cultured pancreatic cancer cells were harvested and lysed with RIPA lysis buffer containing cOmplete protease inhibitor cocktail (04693132001; Roche, Switzerland) and PhosSTOP phosphatase inhibitor (04906845001; Roche, Switzerland). The protein concentration was determined with a BCA protein quantification assay. Proteins in each sample were separated by sodium dodecyl sulfate-polyacrylamide gel electrophoresis and detected on PVDF membranes. The antibodies used for Western blot were as follows: anti-ENO1 (ab227978, 1:1,000; Abcam, USA), anti-caspase-3 (9,662, 1:1,000; CST, China), anti-GAPDH (sc-47724, 1:3,000; Santa Cruz, USA), anti-CA9 (ab243660, 1:1,000; Abcam, USA), anti-ERK (sc-514302, 1:2,000; Santa Cruz, USA), and anti-phospho-Erk1/2 (Thr-202/Tyr-204) (4,370, 1:1,000; CST, China). Blots of the target proteins were detected with WesternBright ECL HRP substrate (R-03031-D2; Advansta, USA) and visualized with a C-DiGit Blot Scanner (LI-COR Biosciences, USA).

### Detection of apoptosis

Apoptotic cells were quantified with an APC Annexin V/propidium iodide (PI) Apoptosis Detection Kit (A6030; US EVERBRIGT) according to the manufacturer’s instructions. Briefly, SW1990 and PANC-1 cells transfected with shENO1 or shNTC were added to 6-well culture plates at 80% confluence and then incubated with 150 μM (SW1990) or 600 μM (PANC-1) CoCl_2_ or vehicle (dd water) for 24 h. Cells harvested with trypsin were stained with 5 μL of APC annexin V and 10 μL of PI for 15 min. Untreated cells were used as negative controls. Data analysis was performed with a BD AccuriR C6 flow cytometer (BD Biosciences). Annexin V-positive and PI-negative cells were identified as early apoptotic cells, whereas Annexin V-positive and PI-positive cells were identified as late apoptotic cells. The extent of apoptosis was calculated as a percentage of both cell populations. The experiments were performed in triplicate.

### RNA-seq and analysis

Total RNA from SW1990 cells transfected with shENO1 and shNTC was harvested with TRIzol reagent and subjected to mRNA-seq by LC-Bio (Zhejiang, China). Venn, GO enrichment, and GSEA was performed by LC-Bio (https://www.lc-bio.cn/).

### Immunofluorescence staining

Pancreatic cancer cells from different groups were seeded onto glass slides. After reaching 70%–80% confluence, the cells were washed with PBS and fixed with 4% paraformaldehyde at room temperature. The cells were then permeabilized with 0.1% Triton X-100 in PBS for 20 min. Nonspecific antigens were blocked with 5% fetal bovine serum in PBS at room temperature for 30 min. The slides were incubated overnight with primary antibodies at 4 °C and subsequently with secondary antibodies for 1 h, then washed with PBS. Next, the nuclei were stained with DAPI (Sigma-Aldrich). The slides were observed and imaged under a confocal microscope (A1; Nikon).

### Tumor engraftment in ischemic hindlimbs of nude mice

The animal experiments were approved by the Ethics Committee of Tianjin Medical University Cancer Institute & Hospital (NFEC-AE-2022032). All steps were carefully performed to protect the welfare of the animals and minimize suffering. Briefly, 5-week-old female nude mice were purchased from Beijing HFK Biosciences (Beijing, China). After 1 week of adaptation, the mice were randomly divided into 2 groups. The mice were anesthetized with 10% chloral hydrate. The skin of the left groin was cut; the femoral artery and its branches were ligated; and the wound was sutured. The transplanted tumors were inoculated 24 h after surgery. Approximately (1–2) × 10^6^ ENO1-sh SW1990 cells, ENO1-sh PANC-1 cells, or control cells were subcutaneously injected into the left groin in the mice (*n* = 6). These tumor cells were grown in an ischemic microenvironment. The same cells were engrafted into the right groin in the mice to control the normal tumor microenvironment. Tumors were measured daily, and tumor volume was calculated with a standard formula (length × width^2^ × 0.52). All mice were sacrificed when the average tumor volume reached 1.0 cm^3^.

### Statistical analysis

Statistical analyses were performed in GraphPad Prism 6 software (GraphPad Software, La Jolla, CA, USA). Data are expressed as mean ± SD. Unpaired Student’s *t*-tests were performed. The threshold for statistical significance was set at *P* < 0.05.

## Results

### Effects of ENO1 expression on the pathological and clinical features of human PDAC

**Figure 1:**
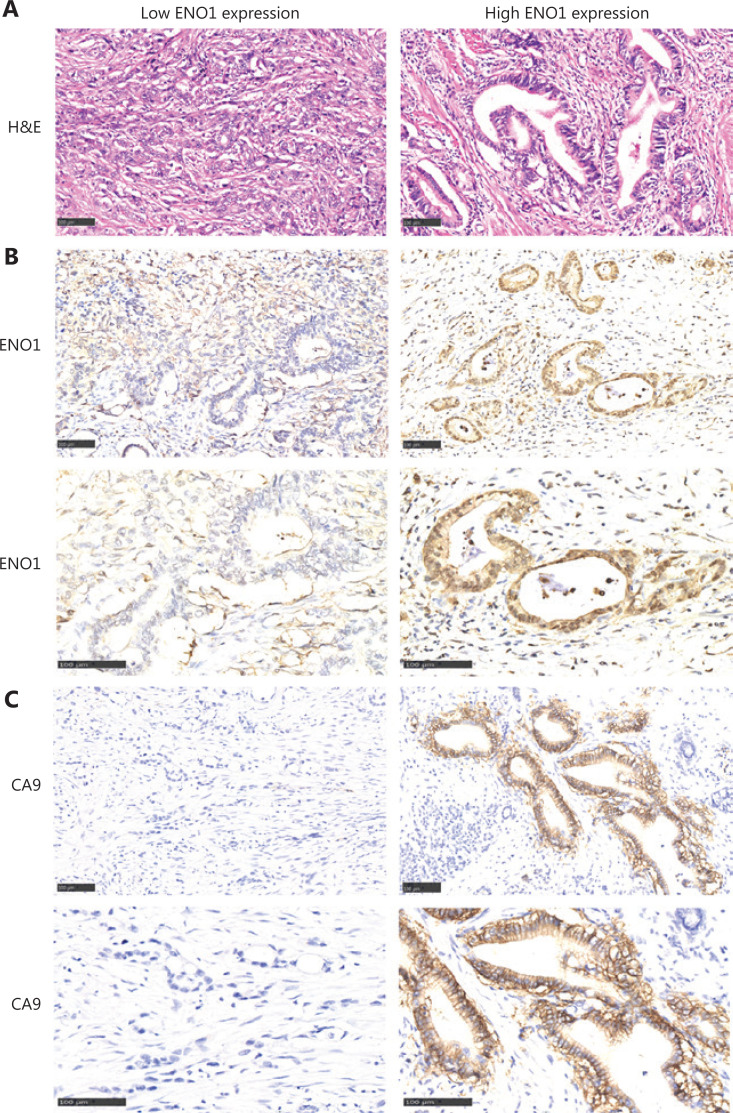
Comparison of histology and ENO1 and CA9 expression in human pancreatic cancer with high and low ENO1 expression. (A) H&E staining indicating histology in human pancreatic cancer with high and low ENO1 expression. (B) ENO1 expression in the high ENO1 group and low ENO1 group. (C) CA9 expression in the high ENO1 group and low ENO1 group. Bar = 100 μm.

In this study, 34 pancreatic cancers expressed ENO1 at high levels, and 50 expressed ENO1 at low levels. ENO1 was expressed mainly in the cytoplasm and nuclei of pancreatic cancer cells, whereas CA9 was expressed in the cell membrane (**Figure 1**). **Supplementary Table S1** compares the pathological and clinical features of patients in the low and high ENO1 expression groups. The percentage of female patients in the low and high ENO1 expression groups was 26.0% and 50.0%, respectively (χ^2^ = 5.077, *P* = 0.002; **Supplementary Table S1**). Approximately 17.6% of patients with pancreatic cancer with high ENO1 levels were younger than 50 years of age, and 6.0% of patients with low ENO1 levels were younger than 50 years of age (χ^2^ = 2.807, *P* = 0.092; **Supplementary Table S1**). More pancreatic cancers with larger tumor diameters were observed in the high ENO1 expression group than in the low ENO1 expression group (χ^2^ = 5.739, *P* = 0.043; **Supplementary Table S1**). Approximately 64.7% of patients with pancreatic cancer with high ENO1 levels showed high-to-moderate differentiation, and 50% of patients with low ENO1 levels showed high-to-moderate differentiation (χ^2^ = 3.552, *P* = 0.042; **Supplementary Table S1**). Tumors in the high ENO1 expression group showed more nerve and vascular invasion than those in the high ENO1 expression group (χ^2^ = 1.976, *P* = 0.111; χ^2^ = 2.282, *P* = 0.099; **Supplementary Table S1**).

The correlations between ENO1 expression and the pathological and clinical features of 84 pancreatic cancer cases were tested with Pearson correlation coefficient analysis (**Supplementary Table S2**). A significant negative correlation was observed between ENO1 expression and patient age at diagnosis (*r* = −0.022, *P* = 0.009; **Supplementary Table S2**). ENO1 expression levels in this study were positively correlated with nerve invasion (*r* = 0.180, *P* = 0.020; **Supplementary Table S2**). The serum levels of tumor markers such as CA19-9, CA242, and CEA were positively correlated with ENO1 expression (**Supplementary Table S2**). ENO1 expression levels were positively associated with the expression of the hypoxia-associated marker CA9 (*r* = 0.126, *P* = 0.039; **Supplementary Table S2**). More CA9-positive pancreatic cancers were found in the high ENO1 expression group than the low ENO1 expression group (**Figure 1**).

### Effects of ENO1 expression on the survival of patients with PDAC

**Figure 2:**
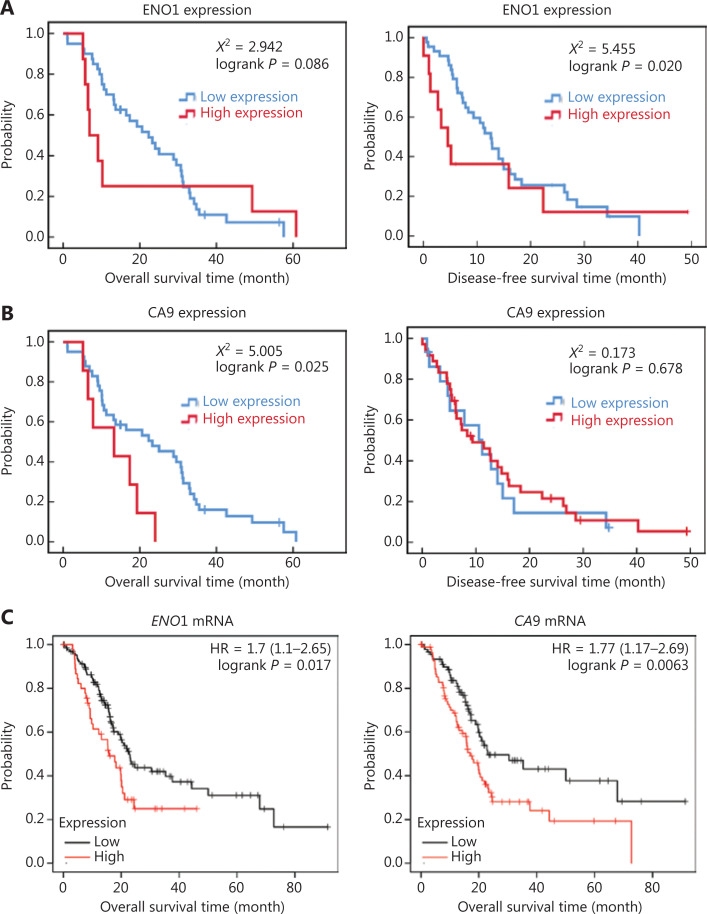
Kaplan-Meier survival plot of ENO1 in human pancreatic ductal adenocarcinoma (PDAC). (A) Overall survival and disease-free survival plot of ENO1 protein levels in 84 PDAC cases. (B) Overall survival and disease-free survival plot of CA9 protein levels in 84 patients with PDAC. (C) Overall survival plot of *ENO1* and *CA9* mRNA expression in 178 PDAC samples from the KM-Plotter database.

The median overall survival of all patients with pancreatic cancer was 16.9 months, and the median disease-free survival time was 9.0 months. The average overall survival time of the high ENO1 expression group was 19.196 ± 7.920 months, whereas that of the low ENO1 expression group was 23.077 ± 2.387 months. The mean disease-free survival time of the high ENO1 expression group was 12.243 ± 4.930 months, whereas that of the low ENO1 expression group was 15.526 ± 1.915 months. Kaplan-Meier analyses revealed that ENO1 expression was associated with poor disease-free survival (χ^2^ = 5.455, *P* = 0.020; **Figure 2A**) but not poor overall survival (χ^2^ = 2.942, *P* = 0.086; **Figure 2A**). Patients with pancreatic cancer with high CA9 expression had poor overall survival (χ^2^ = 5.005, *P* = 0.025; **Figure 2B**).

The KM-Plotter website was used to analyze and evaluate the clinical significance of the findings. According to 178 PDACs in the KM-Plotter database, high expression of ENO1 and CA9 was associated with poor prognosis in patients with PDAC (**Figure 2C**). These results are consistent with the relationship observed in our patient data.

### ENO1 silencing induces PDAC cell apoptosis and other behaviors under hypoxia

**Figure 3:**
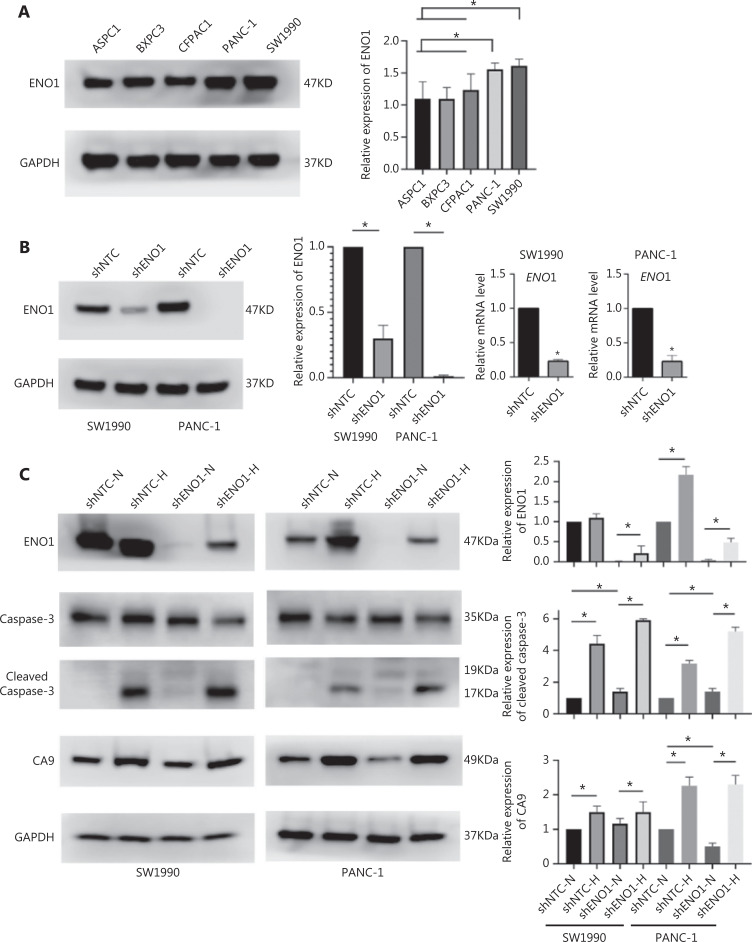
Pancreatic cancer cells with ENO1 gene knockout are more prone to apoptosis in the hypoxia microenvironment. (A) Immunoblotting of ENO1 expression levels in pancreatic cancer cell lines and quantification of ENO1 expression levels. (B) Western blot assays and qRT-PCR for the detection of ENO1 expression in SW1990 and PANC-1 cells with stable ENO1-knockdown. ENO1 expression was successfully abolished by shENO1. Quantification of ENO1 expression levels in these cells is shown. The numbers below the blots are quantitative ratios of the ENO1/GAPDH band densities. shNTC: non-targeting control; shENO1: shRNA against ENO1. (C) Upregulated expression of ENO1 and CA9 under CoCl_2_-induced hypoxia-mimicking conditions in SW1990 and PANC-1 cells with ENO1 silencing. Cells were treated with CoCl_2_ at a concentration of 150 μM (SW1990) or 600 μM (PANC-1) for 24 h. ENO1 silencing and CoCl_2_-induced hypoxia increased the levels of cleaved caspase-3. Quantification of protein expression levels in these cells is shown. *P < 0.05.

**Figure 4:**
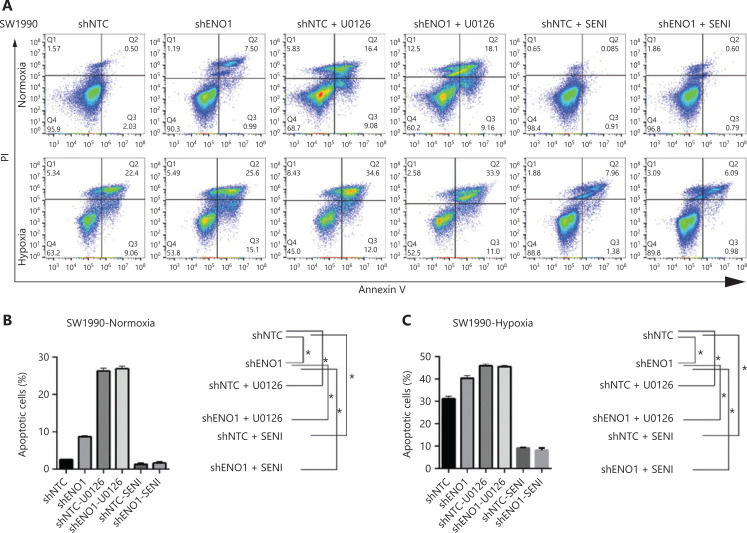

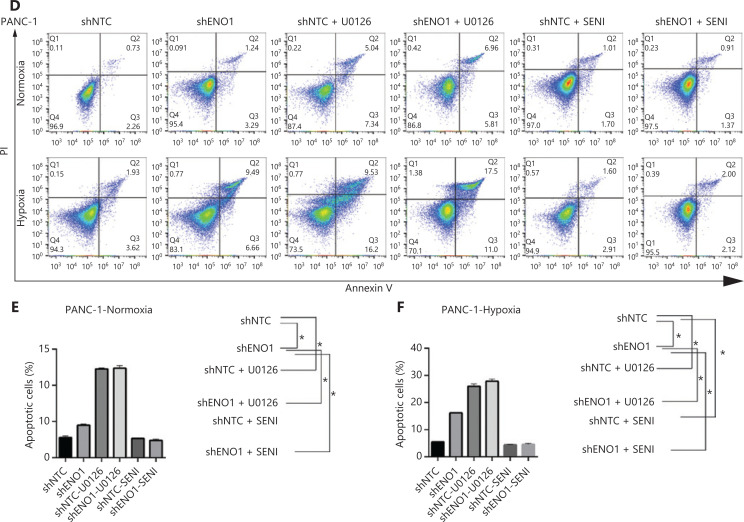
ENO1 knockdown enhances apoptosis by inactivating the ERK pathway in SW1990 and PANC-1 cells. (A–C) Apoptosis of SW1990 cells with the indicated treatments, as measured with Annexin V assays and flow cytometry. The percentage of apoptotic cells is plotted. Annexin V assays were performed 3 times, and values are expressed as the mean ± standard deviation of 3 independent experiments. (D–F) Apoptosis of PANC-1 cells with the indicated treatments, as measured with Annexin V assays and flow cytometry. The percentage of apoptotic cells is plotted. Annexin V assays were performed 3 times, and values are expressed as the mean ± standard deviation of 3 independent experiments. *P < 0.05 as compared with the control group.

To evaluate the expression of ENO1 in the pancreatic cancer cell lines used in this study, we performed Western blot and found that the expression of ENO1 was high in the 5 human PDAC cell lines (**Figure 3A**). Consequently, stable knockdown of ENO1 in SW1990 and PANC-1 cells was achieved through lentiviral delivery of a specific small interfering RNA for ENO1. The efficiency of ENO1 knockdown was validated by Western blot and quantitative reverse-transcription polymerase chain reaction (qRT-PCR). The expression of ENO1 was significantly lower than that of shNTCs (**Figure 3B**). To investigate whether ENO1 has an anti-apoptotic role in pancreatic cancer, we performed Western blot to examine changes in the expression of full-length and cleaved caspase-3 after ENO1 knockdown. The expression of cleaved caspase-3 was significantly higher in shENO1 cells than control cells (**Figure 3C**). Furthermore, annexin V-FITC/PI staining analysis showed that the apoptotic cell percentage was higher in the ENO1-silenced group than the shNTC group (**Figure 4A–4F**).

Because the ENO1 promoter contains a hypoxia-responsive element, significant increases in ENO1 levels were found in shENO1 cells after 24 h of CoCl_2_ treatment. CA9 protein levels were also elevated, thus indicating that ENO1 expression was successfully induced by CoCl_2_-induced hypoxia. Apoptosis assays revealed that ENO1 silencing accelerated apoptosis under CoCl_2_-induced hypoxia (**Figure 3C**). These results demonstrated that ENO1 knockdown induced apoptosis under both normoxic and hypoxic conditions.

We also detected the effects of CoCl_2_-induced hypoxia and ENO1 downregulation on the migration, invasion, and proliferation of human PDAC cells. Hypoxia and ENO1 downregulation inhibited the migration, invasion, and proliferation of SW1990 and PANC-1 cells (**Supplementary Figures S1–S3**).

### RNA-seq reveals ENO1-associated apoptosis and signaling pathways

**Figure 5:**
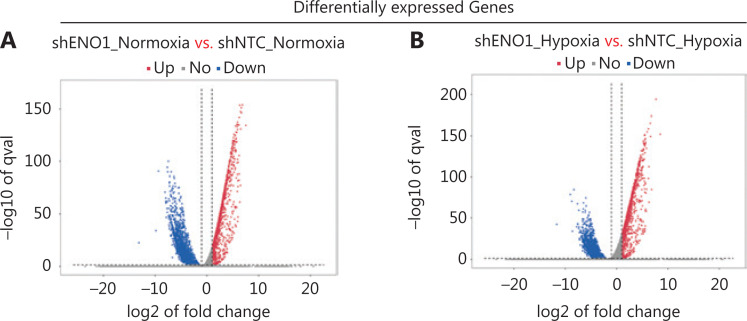

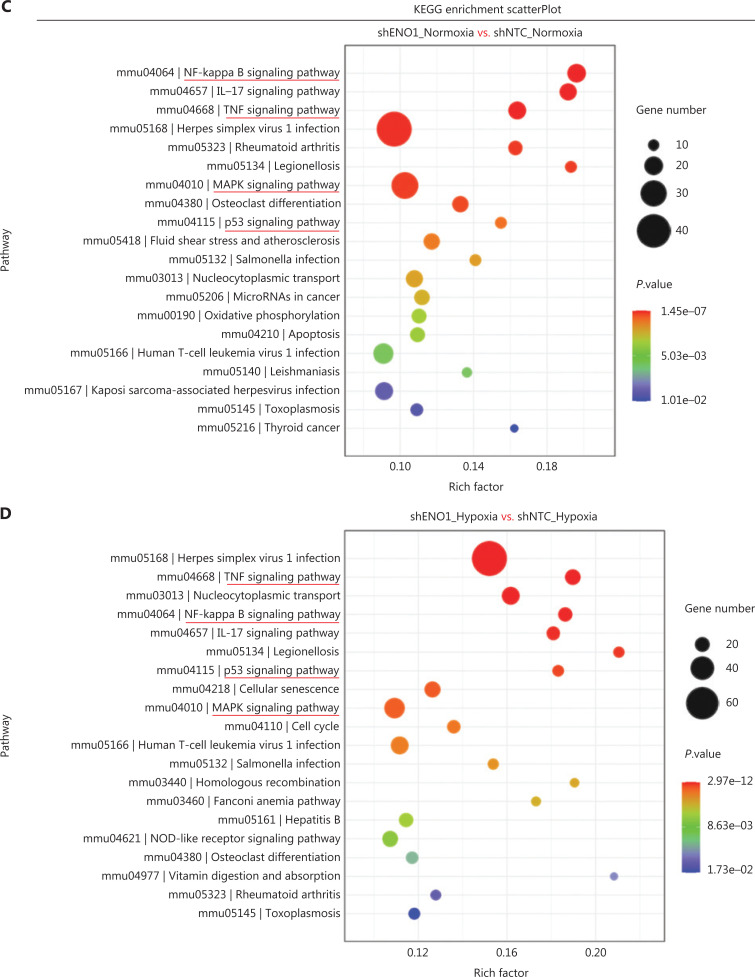

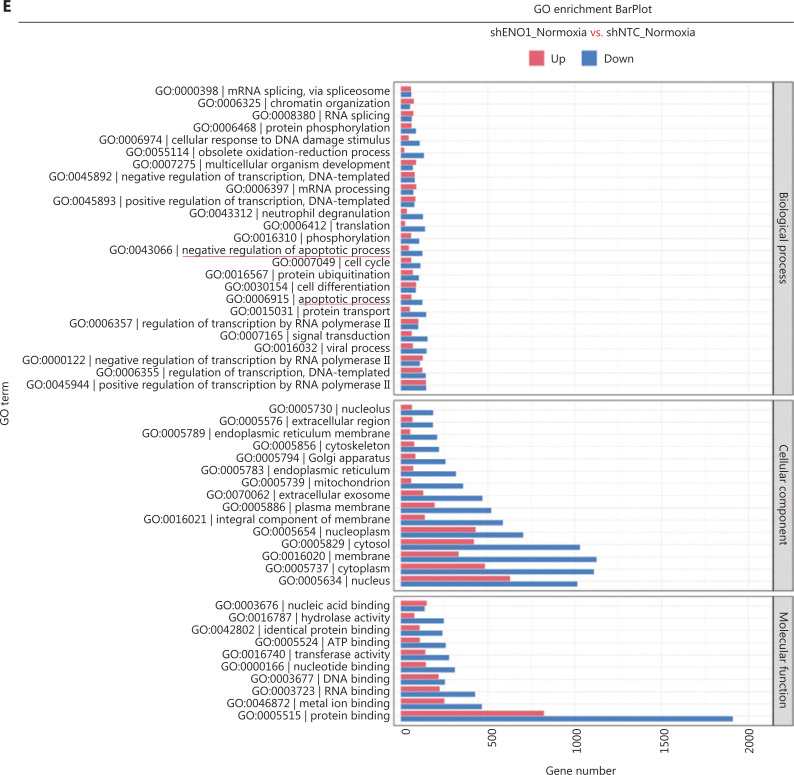

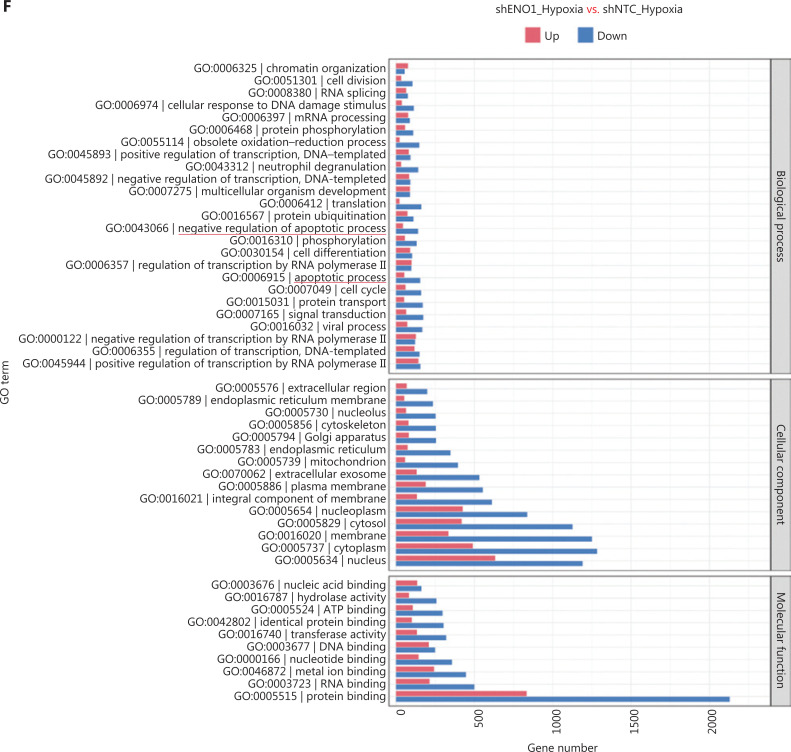
RNA-seq analysis of ENO1-knockdown cells. (A) and (B) Volcano plots demonstrating the differentially expressed genes (DEGs) (fold-change >2 and false discovery rate <0.001) regulated by ENO1 knockdown in SW1990 cells. (C) and (D) KEGG analysis of signaling pathways regulated by ENO1 knockdown in SW1990 cells. (E) and (F) GO enrichment analysis of DEGs, divided into 3 functional groups: biological process (BP), cellular component (CC), and molecular function (MF).

To assess the effects of ENO1 silencing and CoCl_2_-induced hypoxia on pancreatic cancer transcriptomes, we extracted total RNA from SW1990-shENO1 and SW1990-shNTC cells treated with either CoCl_2_ or distilled water for 24 h, then performed RNA sequencing analysis. We then analyzed and focused on differentially expressed genes (DEGs) of shENO1_N *vs.* shNTC_N (N: normoxia), in which 1,141 genes were upregulated, and 2,430 genes were downregulated, and DEGs of shENO1_H *vs.* shNTC_H (H: hypoxia), in which 1,143 genes were upregulated, and 2,653 genes were downregulated (**Figure 5A, 5B**). GSEA and GO analysis indicated that ENO1 knockdown regulated various biological processes, specifically those associated with the negative regulation of apoptotic processes (**Figure 5C–5F** and **Supplementary Figure S4**). Moreover, KEGG pathway analysis indicated that the transcription factor nuclear factor κB (NF-κB), tumor necrosis factor (TNF), P53, and mitogen-activated protein kinase (MAPK) signaling pathways were markedly enriched in ENO1-knockdown cells under both normoxia and hypoxia (**Figure 5C–5F**).

### ENO1 mediates apoptosis by regulating ERK activation

**Figure 6:**
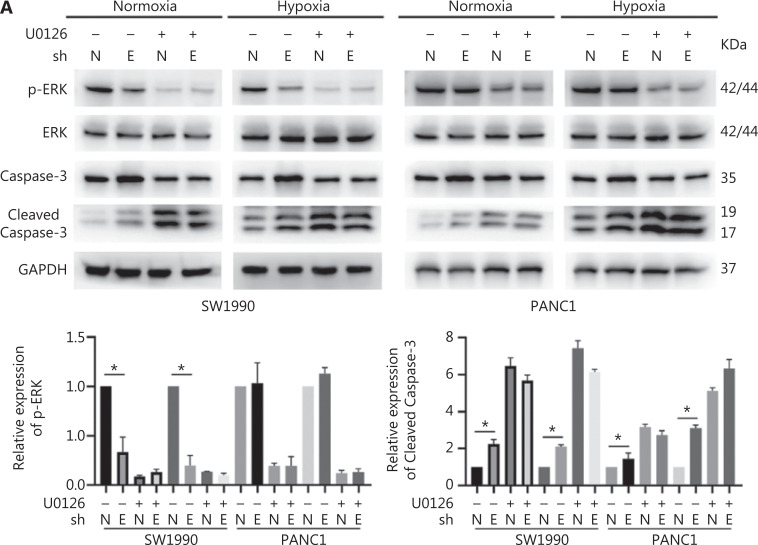

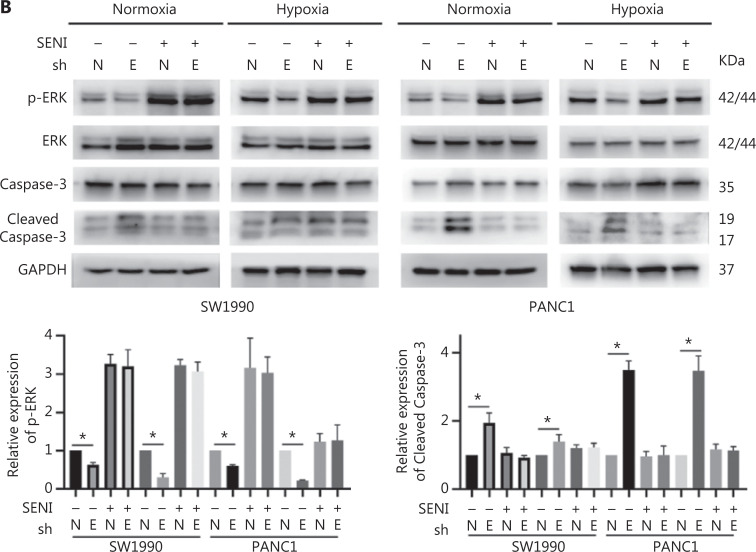

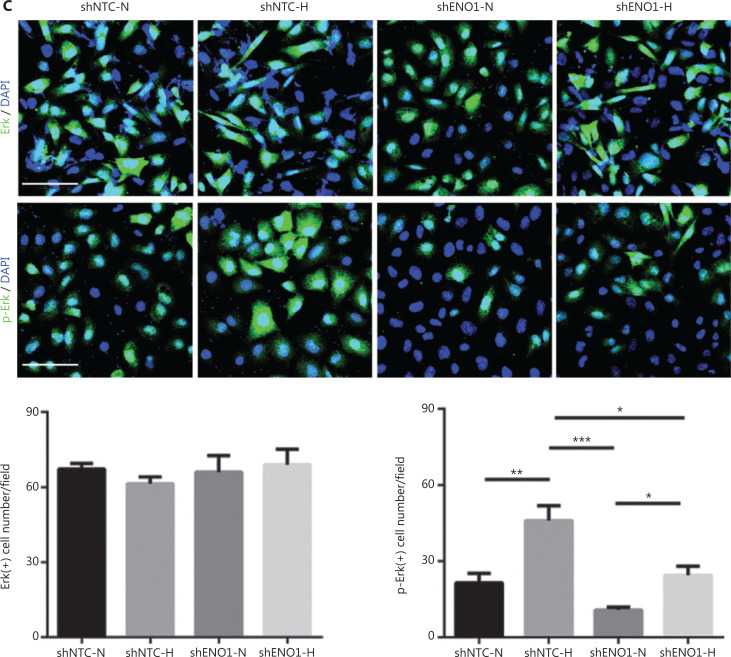
ENO1 knockdown enhances apoptosis by inactivating the ERK pathway. (A) and (B) Immunoblotting of ENO1, ERK, p-ERK (Thr202/Tyr204), and caspase-3 with an ERK inhibitor or activator in normoxia and hypoxia. The values under each lane indicate the relative density of the band normalized to that of GAPDH, according to densitometry. Quantification of protein expression levels in these cells. (C) Immunofluorescence illustrating ERK and p-ERK expression in PANC-1-shNTC and PANC-1-shENO1 cells. Quantification of ERK and p-ERK expression levels in these cells. Scale bars = 50 μm. *P < 0.05, **P < 0.01, ***P < 0.005.

To confirm the above enriched pathways, we examined the effects of ENO1 knockdown on the NF-κB and MAPK signaling pathways through Western blot. ENO1 knockdown significantly decreased the phosphorylation levels of ERK, but not p38 or c-Jun N-terminal kinase (JNK); moreover, the total ERK, p38, and JNK protein levels were unchanged (**Figure 6A, 6B, and Supplementary Figure S5**). To explore whether ERK inactivation was associated with cell apoptosis, we next detected the total protein and phosphorylation levels of ERK1/2 under both normoxic and hypoxic conditions, after treatment with a MEK1/2 inhibitor (U0126, 10 μM) or ERK agonist (SENI, 5 μM). As shown in **Figures 6B and 4A–4F**, the MEK1/2 inhibitor (U0126) enhanced the effect of ENO1 downregulation on ERK1/2 activity and cell apoptosis. These effects were reversed when the cells were incubated in the presence of the EKR1/2 activator. Immunofluorescence findings illustrating ERK and p-ERK expression in PANC-1-shNTC and PANC-1-shENO1 cells confirmed the results (**Figure 6C**). These data indicated that the anti-apoptotic role of ENO1 is mediated by ERK activation.

### ENO1 mediates hypoxia-induced growth of pancreatic cancer cells transplanted into ischemic hindlimbs of mice

**Figure 7:**
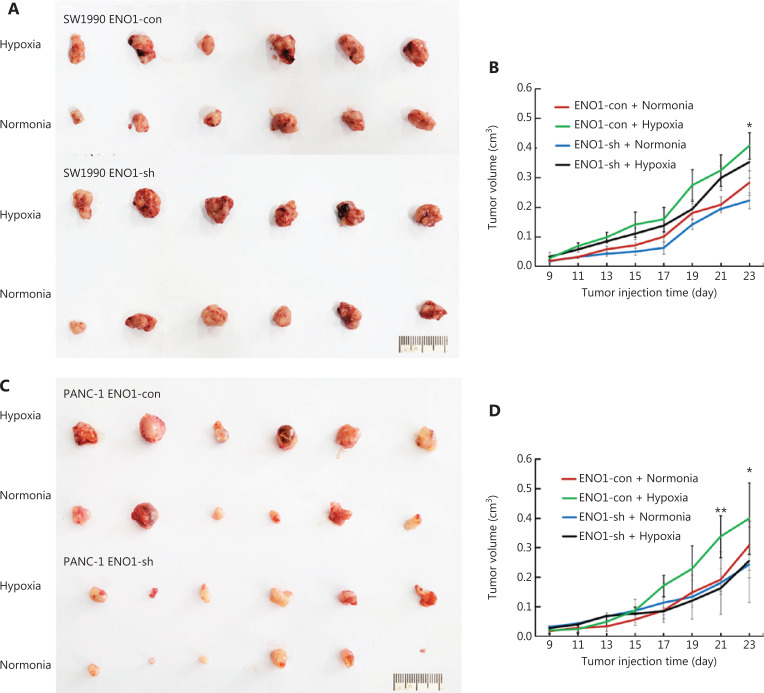
Growth of transplanted pancreatic tumor cells in ischemic and control hindlimbs of nude mice. (A) and (B) Growth of transplanted SW1990-shENO1 and SW1990-con tumor cells in ischemic and control nude mice. (C) and (D) Growth of PANC-1-shENO1 and PANC-1-con transplanted tumor cells in ischemic and control hindlimbs of nude mice. *P < 0.05, **P < 0.01.

To investigate whether ENO1 might be involved in the growth of pancreatic cancer in a hypoxic microenvironment, we established a nude mouse hindlimb model to simulate the hypoxic microenvironment *in vivo*. SW1990-shENO1 and PANC-1-shENO1 human pancreatic cancer cells, and controls were injected into the ischemic hindlimbs of the mice. Tumors in the control SW1990 and PANC-1 cells grew faster in the hypoxic microenvironment than in the normoxic microenvironment (**Figure 7A–7D**). No significant difference in tumor growth of SW1990-shENO1 and PANC-1-shENO1 cells was observed between hypoxic and normoxic microenvironments (**Figure 7A–7D**). Further studies indicated no significant difference in the proportion of necrotic areas in the tumor tissues of each group; however, the proportion of poorly differentiated areas in ENO1-knockdown PDAC cells increased significantly in the hypoxic microenvironment (**Supplementary Figure S6A**).

**Figure 8:**
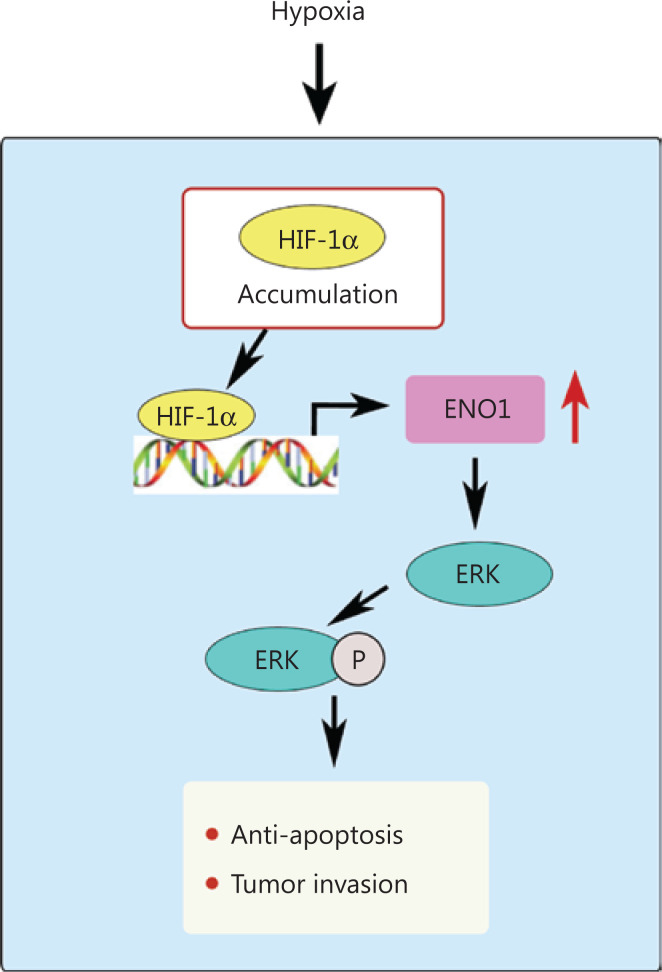
Hypoxia increases the expression of ENO1 in pancreatic cancer cells. ENO1 promotes ERK phosphorylation in the MAPK pathway and protects pancreatic cancer cells from apoptosis, thereby increasing the survival and invasion of pancreatic cancer cells in the hypoxic microenvironment.

Interestingly, comparison of the expression of the hypoxia-associated molecule CA9 in each group demonstrated that the hypoxic microenvironment stimulated CA9 expression in ENO1-con pancreatic cancer cells but had no effect in the ENO1-knockdown group (**Supplementary Figures S6A–S6F and S7A, S7B**). This finding indicated that the upregulation of the HIF-1α pathway in the hypoxic microenvironment is mediated by ENO1. To study the mechanism of enhanced tumor growth after ENO1 activation in a hypoxic microenvironment, we determined the cell proliferation and apoptosis rates in each group of tumors. Immunohistochemical staining for Ki-67 showed that the proliferation activity of tumor cells in the well-differentiated area of the ENO1-con group significantly increased in the hypoxic microenvironment, whereas no significant change was observed in the proliferation ability of tumor cells in the well-differentiated area of the ENO1-knockdown groups (**Supplementary Figures S6A–S6F and S7A, S7B**). The proliferation of poorly differentiated tumor cells in each group was very strong. TUNEL staining indicated that the apoptosis of pancreatic cancer cells increased in the hypoxic microenvironment, whereas that in the ENO1-con group was significantly lower than that in the ENO1-sh group (**Figure 8E–8F**). These results suggested that hypoxia-induced ENO1 expression partially inhibits tumor cell apoptosis.

## Discussion

ENO1 has been reported to regulate tumorigenesis and the development of various tumors. Many studies have shown that ENO1 is associated with pancreatic invasion and metastasis^11^. ENO1 is expressed in human pancreatic cancer cell lines, pancreatic cancer samples, and pancreatic cancer^13^. Yin et al.^14^ have collected 31 specimens of human pancreatic cancer and found elevated ENO1 expression in the tumor tissues. In this study, 84 human pancreatic cancer samples were collected—a larger number than examined in previous studies. ENO1 expression in human pancreatic cancer cells was detected with immunohistochemical staining. The prognosis of patients with high ENO1 expression was poor. These results were confirmed through an online database on pancreatic cancer. The prognosis of pancreatic cancer in the high ENO1 expression group was poorer than that in the low expression group. The expression levels of ENO1 in human pancreatic cancer are closely associated with those of the hypoxia marker CA9 as well as nerve and vascular invasion^12,15^, thus suggesting that ENO1 promotes the invasion and metastasis of pancreatic cancer, in agreement with the results of many *in vitro* studies. We also confirmed that the expression of ENO1 correlated with serum CA199 levels^14^, thereby suggesting that the elevated serum ENO1 levels in pancreatic cancer originated from the tumor cells themselves.

Interestingly, high expression of ENO1 was associated with high differentiation of human pancreatic cancer, whereas poorly differentiated pancreatic cancer cells did not express ENO1. Studies in animal models have also indicated that high expression of ENO1 blocks the dedifferentiation of SW1990 cells (highly to moderately differentiated cells) caused by hindlimb ischemia^16^. However, this phenomenon has not been observed in poorly differentiated PANC-1 human pancreatic cancer cell xenografts^16^. This may be associated with the inhibition of pancreatic cancer stem cells by ENO1. However, ENO1 has been found to increase the stemness of gastric cancer stem cells by enhancing glycolysis^17^. These results suggest that ENO1 has different effects on cancer stem cells among tumor tissues. The underlying mechanisms must be further studied in the future.

Pancreatic cancers show substantial tumor hypoxia, in contrast to the adjacent normal pancreas, which shows normal oxygenation^18^. Hypoxic cancers exhibit elevated glycolysis and glycolytic enzymes, including ENO1, which is involved in the Warburg effect^19^. ENO1 is an HIF-1α-regulating target^20^. Hypoxia response elements in ENO1 gene promoters contain essential binding sites for HIF-1α^21^. ENO1 has been reported to sustain proliferation and inhibit apoptosis in multiple types of malignant tumors. In this study, through biochemical and functional approaches, we revealed that ENO1 participates in regulating apoptosis in PDAC cells under CoCl_2_-induced hypoxia. Moreover, the detection of caspase-3 activity and the percentage of apoptotic cells (Annexin V-PI assays) in the shENO1 group supported the anti-apoptotic role of ENO1 in pancreatic cancer cells under normoxia and hypoxia. These results suggested that hypoxia-induced ENO1 upregulation protects pancreatic cancer cells from apoptosis.

To study the molecular mechanism through which the hypoxic microenvironment affects tumors, a variety of experimental methods simulating the hypoxic microenvironment *in vitro* have been developed^22^. CoCl_2_-induced chemical hypoxia is one of the most commonly used hypoxia models in cell culture. CoCl_2_ strongly stabilizes HIF-1α and HIF-2α, and induces the mRNA expression of HIF-1α and HIF-2α under normoxic conditions^23^. However, some differences in biological effects have been observed between CoCl_2_-induced chemical hypoxia and hypoxic conditions^22^. Zhigalova et al.^24^ have found that CoCl_2_-induced hypoxia does not affect glycolysis and the gluconeogenesis pathway, the most common pathways activated by hypoxia. CoCl_2_ and hypoxia induce similar responses in the expression of genes encoding glycolytic enzymes—such as aldolase, phosphoglycerate kinase 1 and pyruvate kinase M—and cell apoptosis^25,26^. Hence, that model was used to study the effect of hypoxia on the apoptosis of pancreatic cancer cells, and the experimental results were reliable.

Mechanistically, the functional relationship between ENO1 and apoptosis has not been thoroughly examined. Previous studies have shown that ENO1 silencing induces Bax, diminishes Bcl-2, and decreases phosphorylation levels of PI3K and AKT in breast cancer; ENO1 silencing results in cell cycle arrest in gastric cancer cells; ENO1 silencing attenuates MAPK activity of p38-MAPK in lung cancer cells; and ENO1 upregulation induces apoptosis in neuroblastoma^27–30^. Thus, to identify the related biological signaling pathways potentially involved in ENO1-mediated apoptosis in PDAC cells, we performed KEGG pathway analysis with RNA-seq. The data indicated that the TNF, NF-κB, and MAPK signaling pathways were markedly enriched in ENO1-silenced cells under both normoxia and hypoxia (**Figure 5**). Studies have shown that tumor necrosis factor receptor-associated factors mediate downstream signaling, including that by NF-κB and MAPKs; therefore, crosstalk among the TNF, NF-κB, and MAPK pathways may be required for ENO1-associated cell fate determination^31^. We then verified the effects of ENO1 silencing on the NF-κB and MAPK signaling pathways, which are strongly associated with hypoxia. Knockdown of ENO1 blocked ERK activity, but not JNK, p38, or p65 activity.

The MAPK signaling pathway is involved in the regulation of cellular proliferation, differentiation, and apoptosis^32^. This pathway consists of 3 main modules: ERK, JNK, and p38 MAPK^33^. In most cases, ERK has a protective effect, whereas JNK and p38 MAPK have pro-death functions^34,35^. We detected the activation of ERKs upon CoCl_2_-induced hypoxia and found decreased ERK activity after ENO1 silencing in pancreatic cancer cells, in agreement with previous findings^36^. Increasing ERK activation with an activator (SENI) and decreasing ERK activation with a MEK1/2 inhibitor (U0126) attenuated and strengthened ENO1 silencing-associated lethality, respectively. In agreement with findings from previous studies^36^, we observed no changes in JNK and p38 MAPK activity. The mechanism of decreased ERK activity induced by ENO1 knockdown may be explained by increased glycolytic signaling and lactic acid production, which in turn induce ERK activation^37^. These findings strongly suggest a functional role of MAPK/ERK signaling in ENO1-mediated anti-apoptotic activity.

The hypoxic tumor microenvironment regulates glucose metabolism in tumor cells and is closely associated with tumorigenesis and tumor development. Numerous studies have implicated hypoxia-dependent/independent pathways and ENO1 upregulation in hypoxic therapeutic resistance^38,39^. Research has focused on the role of ENO1 in tumor progression and cancer treatment^40^. Our data demonstrated that knockdown of ENO1 promotes apoptosis *in vitro* and *in vivo*, and ERK pathway activation blocks this effect. Targeting ENO1 and its downstream ERK phosphorylation therefore may be a promising approach for countering the therapeutic resistance developed during pancreatic cancer growth.

## Conclusions

We found that the prognosis of patients with high ENO1 expression was poor, and ENO1 expression was positively correlated with hypoxia, nerve invasion, and vascular invasion in 84 human pancreatic cancer samples. The CoCl_2_-induced hypoxia model and transcriptomic analysis confirmed that hypoxia increased the expression of ENO1 in pancreatic cancer cells. ENO1 upregulation blocked apoptosis by affecting the MAPK pathway and accelerating ERK phosphorylation. Consequently, survival and invasion of pancreatic cancer cells in the hypoxic microenvironment were promoted (**Figure 8**). Consistent results were obtained in a mouse ischemia model with hindlimbs transplanted with pancreatic tumor cells. ENO1 can be used as a therapeutic target for inhibiting the proliferation, migration, and invasion of pancreatic cancer cells, and for improving the survival of patients with pancreatic cancer.
