# Supplementary material for: ENO1 expression and Erk phosphorylation in PDAC and their effects on tumor cell apoptosis in a hypoxic microenvironment
Source: Cancer Biol Med. 2022 Dec 5;19(11):1598–616. doi: 10.20892/j.issn.2095-3941.2022.0451 (PMC9724225; doi:10.20892/j.issn.2095-3941.2022.0451)
Supplement: Supplementary file 1 [file cbm-19-1598-s001.pdf]

Supplementary materials

Supplementary materials and methods

Wound-healing assays

Cells in all groups were seeded in 6-well plates. When cells reached confluency, a wound was created with a 100-lL sterile pipette tip and photographed (0 h). The rate of gap closure was measured at 24 h and 48 h. Each experiment was performed 3 times.

Cell invasion and cell migration assays

Migration assays were performed in 24-well plates on ENO1-transfected PDAC cells that were added to the upper chamber with serum-free medium, with DMEM with 10% FBS added to the bottom chamber. After the cells were incubated for 24 hours, they were fixed with methanol and stained with crystal violet for 20 minutes. Invasion assays were performed as with the migration assays, except that the Transwell chambers were coated with Matrigel before the cells were seeded in the upper chamber. These cells were counted under an inverted light microscope (Nikon). Each experiment was performed 3 times.

Plate clone formation experiments

The cells were digested according to standard procedures, and the cell suspension was prepared. The concentration was adjusted to 10^5 cells/mL. Approximately, 30 μL cell suspension was seeded into each well of a 6 well plate, which was then placed in an incubator. For observation of clones, the cells were fixed with cold methanol for 20 min and stained with crystal violet. The clones in each group were observed, counted, and imaged under an inverted light microscope (Nikon).

qRT-PCR

Total RNA of SW1990-shENO1, SW1990-shNTC, PANC-1-shENO1, and PANC-1-shNTC cells was isolated with an RNA easy fast tissue/cell kit (DP451, TIANGEN, China) and reverse transcribed with PrimeScript RT Master Mix (RR036A, TaKaRa Biotechnology Co., Ltd). qRT-PCR was performed with a real-time fluorescence quantitative PCR instrument (ABI7500, Thermo Fisher, U.S.A.) with primers as follows: human ENO1 forward primer 5′-CCTGCCCTGGTTAGCAAGAA-3′ and reverse primer 5′-GGCGTTCGCACCAAACTTAG-3′. Human GAPDH was the control, and the primers were as follows: forward primer 5′-TTGCCCTCAACGACCACTTT-3′ and reverse primer 5′-TGGTCCAGGGGTCTTACTCC-3′.

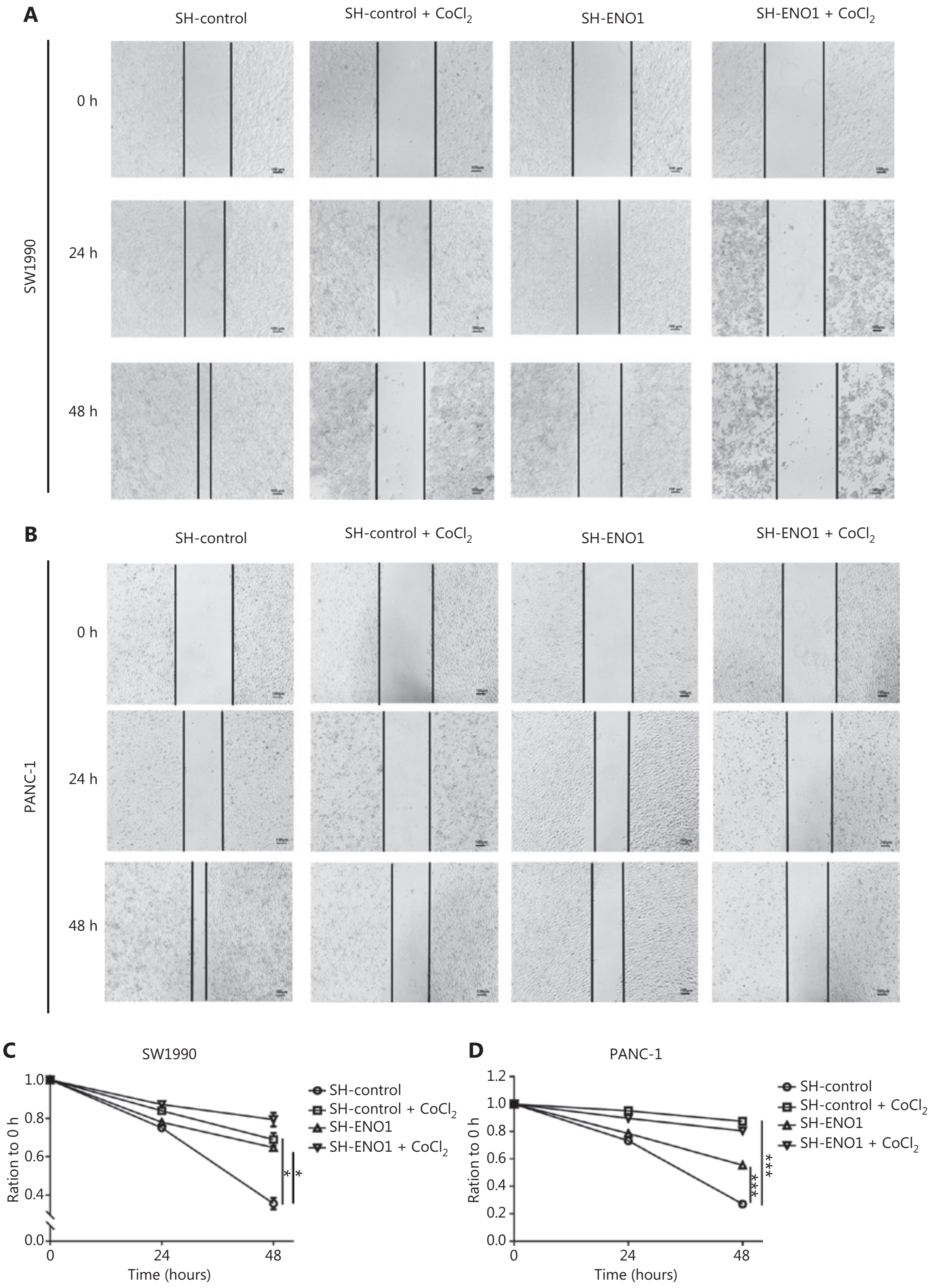

Figure S1 Wound-healing assays on ENO1-transfected cells. (A) Wound-healing assays conducted on SW1990 ENO1-transfected cells. (B) Wound-healing assays conducted on PANC-1 ENO1-transfected cells. (C) Quantification of wound-healing assay results for SW1990 ENO1-transfected cells. (D) Quantification of wound-healing assay results for PANC-1 ENO1-transfected cells. Data are presented as means ± SD. Each experiment was performed in 3 individual replicates. Bars = 100 µm. *$P < 0.05$, ***$P < 0.005$.

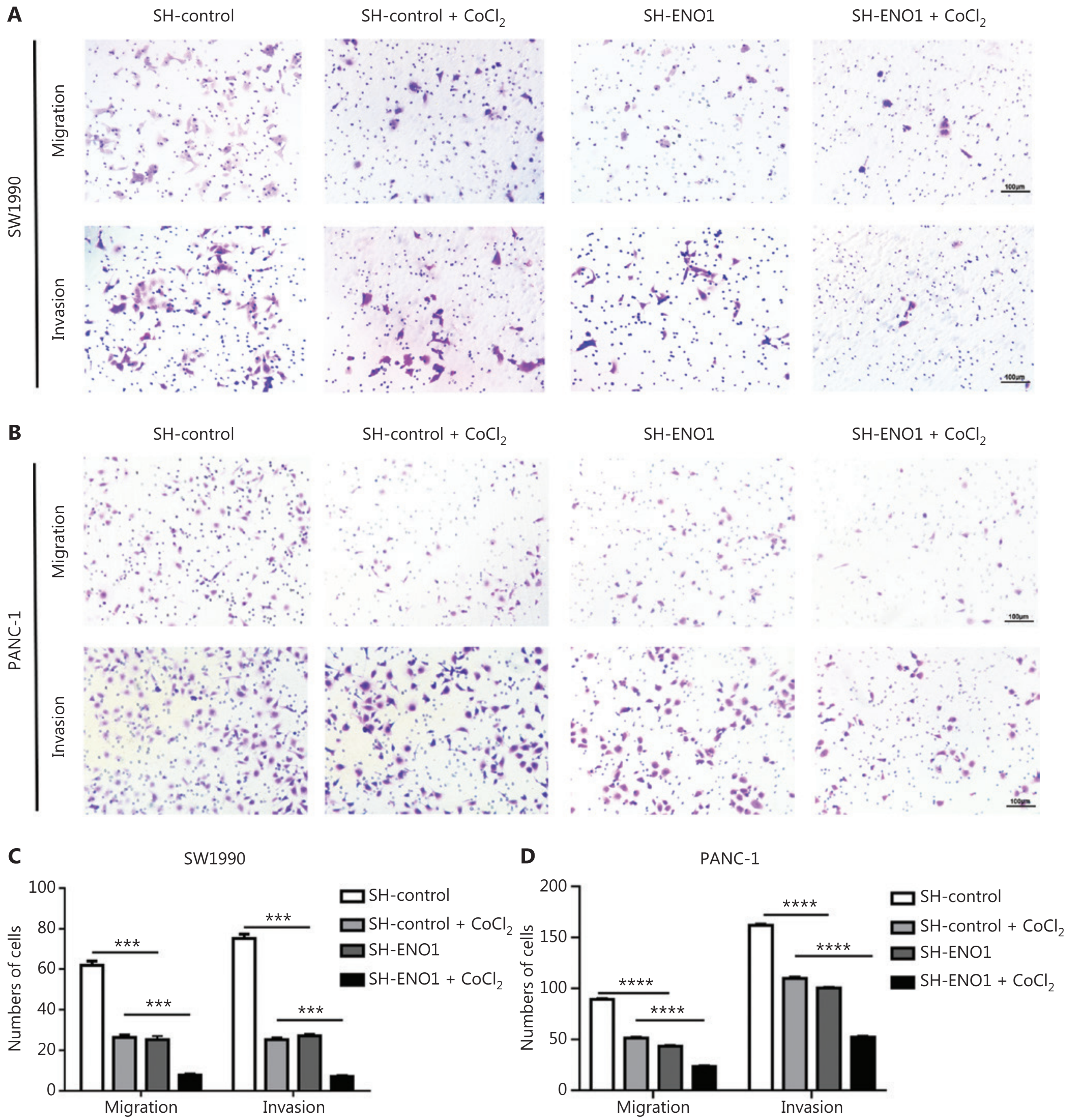

Figure S2 Migration and invasion assays of ENO1-transfected cells. (A) Migration and invasion assays conducted for SW1990 ENO1-transfected cells. (B) Migration and invasion assays conducted for PANC-1 ENO1-transfected cells. (C) Quantification of migration and invasion assay results for SW1990 ENO1-transfected cells. (D) Quantification of migration and invasion assay results for PANC-1 ENO1-transfected cells. These data are presented as means ± SD. Each experiment was performed in 3 individual replicates. Bars = 100 µm. ***P < 0.005, ****P < 0.001.

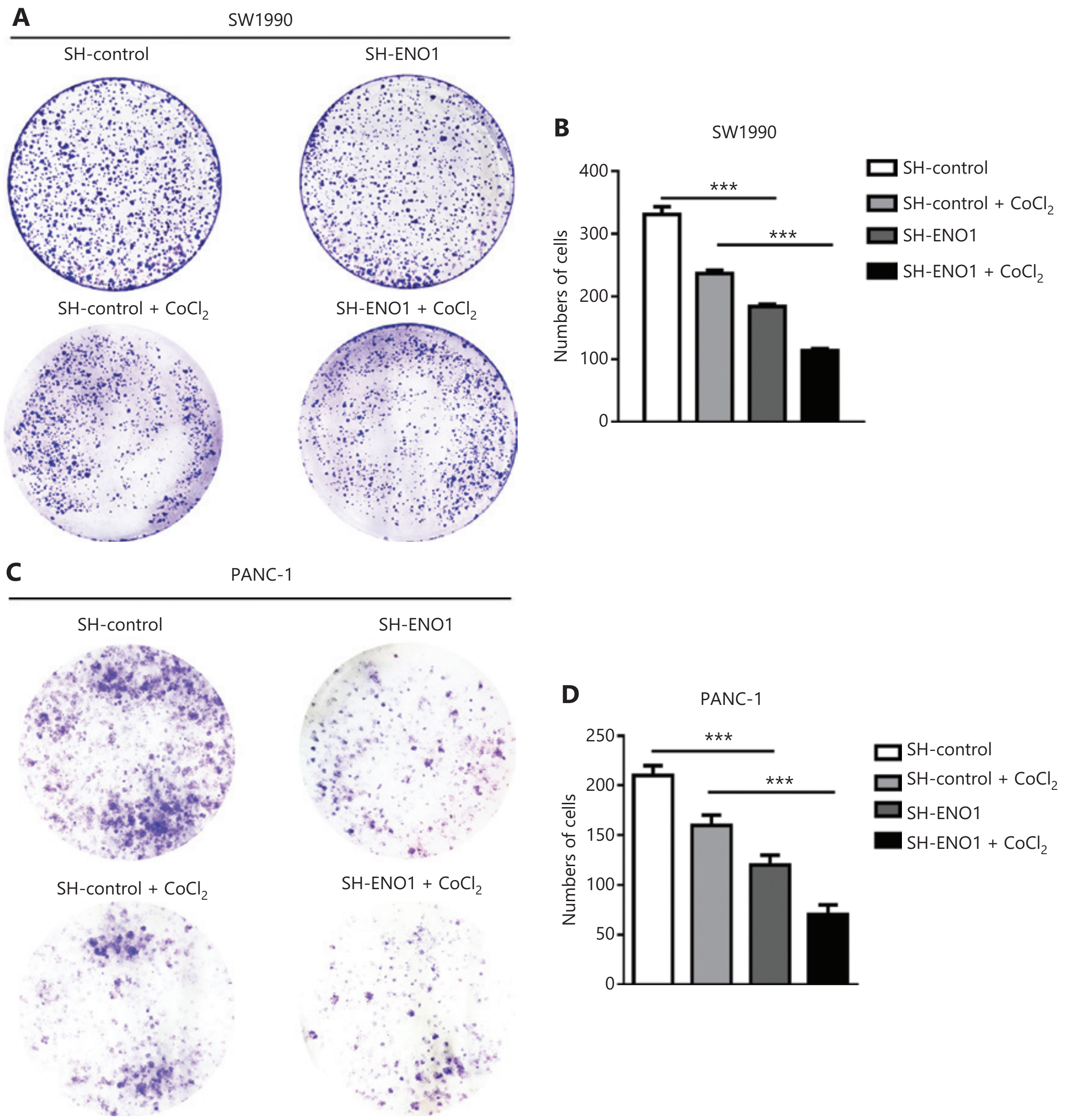

Figure S3 Clone formation assays on ENO1-transfected cells. (A) Clone formation assay conducted for SW1990 ENO1-transfected cells. (B) Clone formation assay conducted for PANC-1 ENO1-transfected cells. (C) Quantification of clone formation assay results for SW1990 ENO1-transfected cells. (D) Quantification of clone formation assay results for PANC-1 ENO1-transfected cells. Data are presented as means ± SD. Each experiment was performed in 3 individual replicates. Bars = 100 µm. ***P < 0.005.

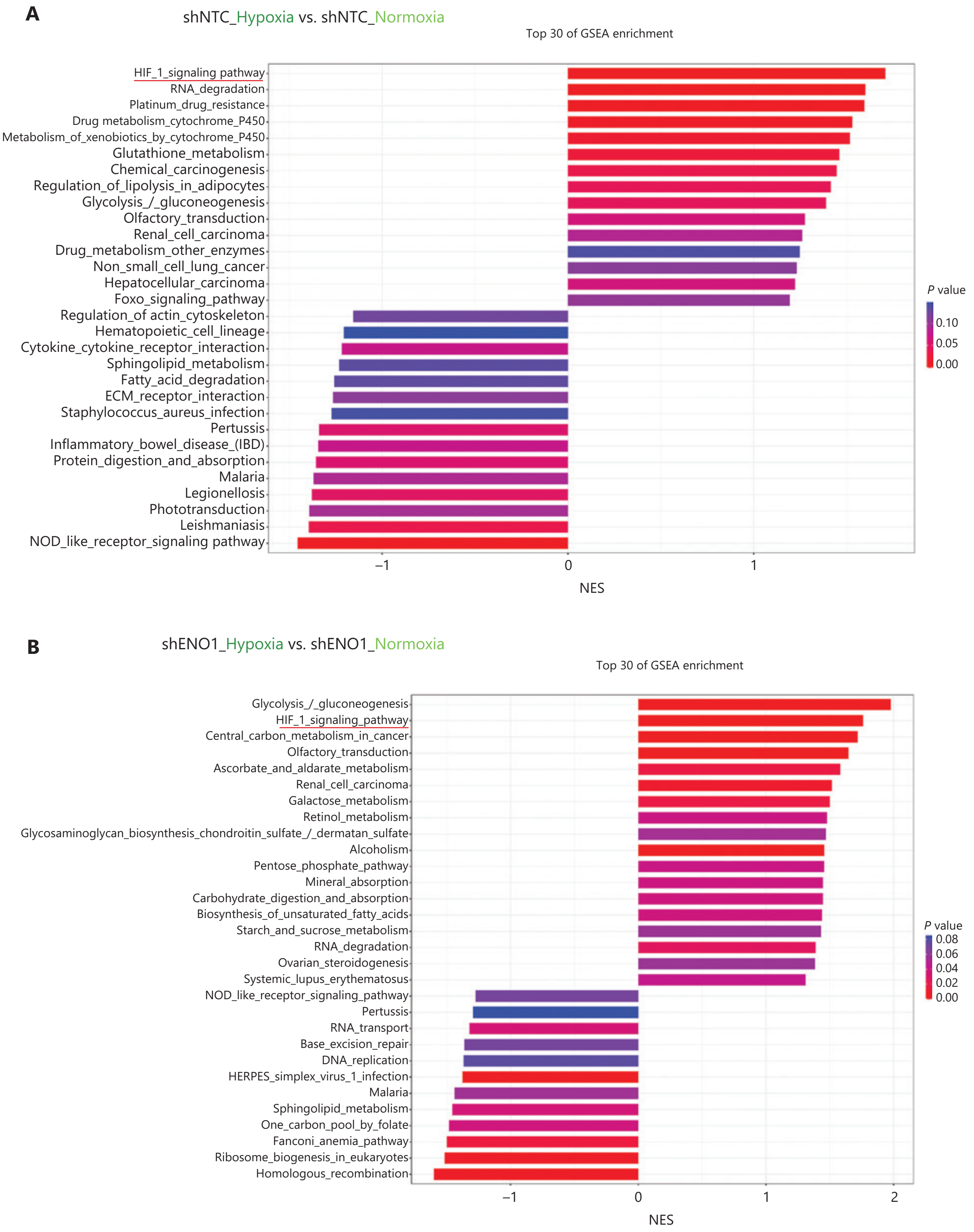

Figure S4 GSEA of signaling pathways regulated by ENO1 knockdown in SW1990 cells. (A) Comparison of signaling pathways between SW1990-shNTC in hypoxic conditions and SW1990-shNTC in normoxic conditions. (B) Comparison of signaling pathways between SW1990-shENO1 in hypoxic conditions and SW1990-sh ENO1 in normoxic conditions.

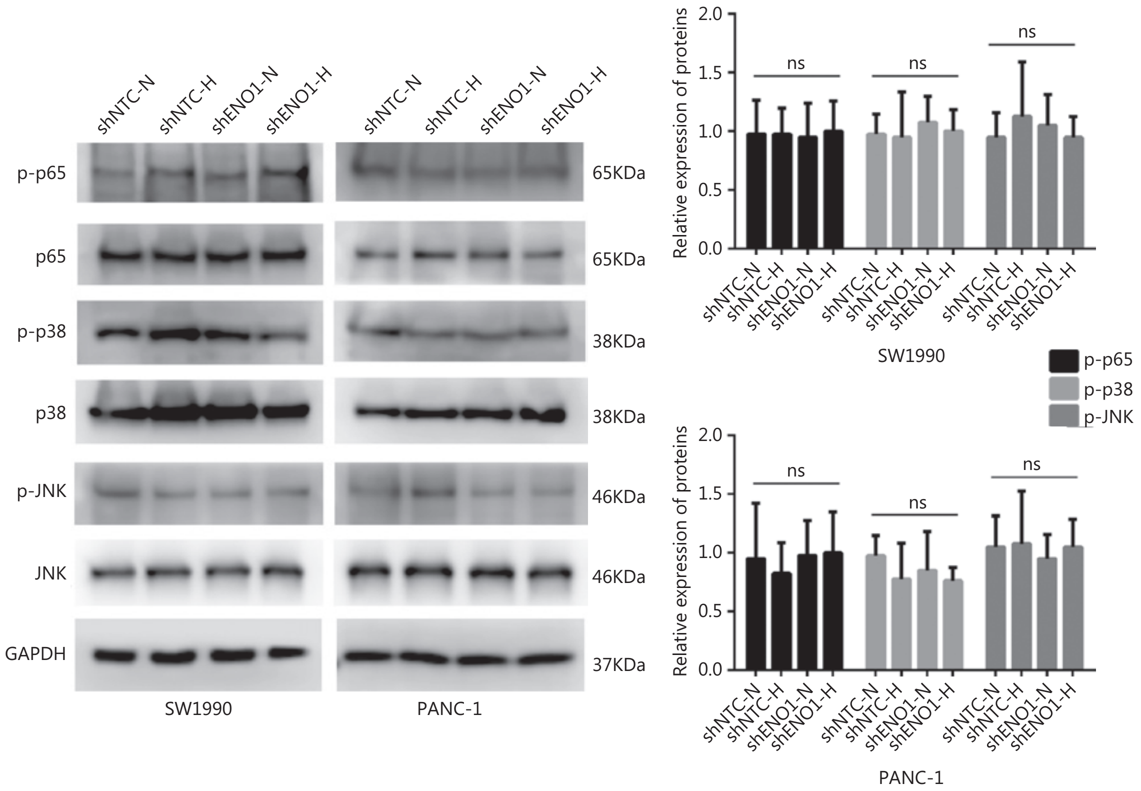

Figure S5 Immunoblotting assays detecting the relative protein levels of p65, p-p65, p38, and p-p38 in SW1990 and PANC-1 cells with stably silenced ENO1. GAPDH was used as the internal control. Quantified protein expression levels in these cells is shown.

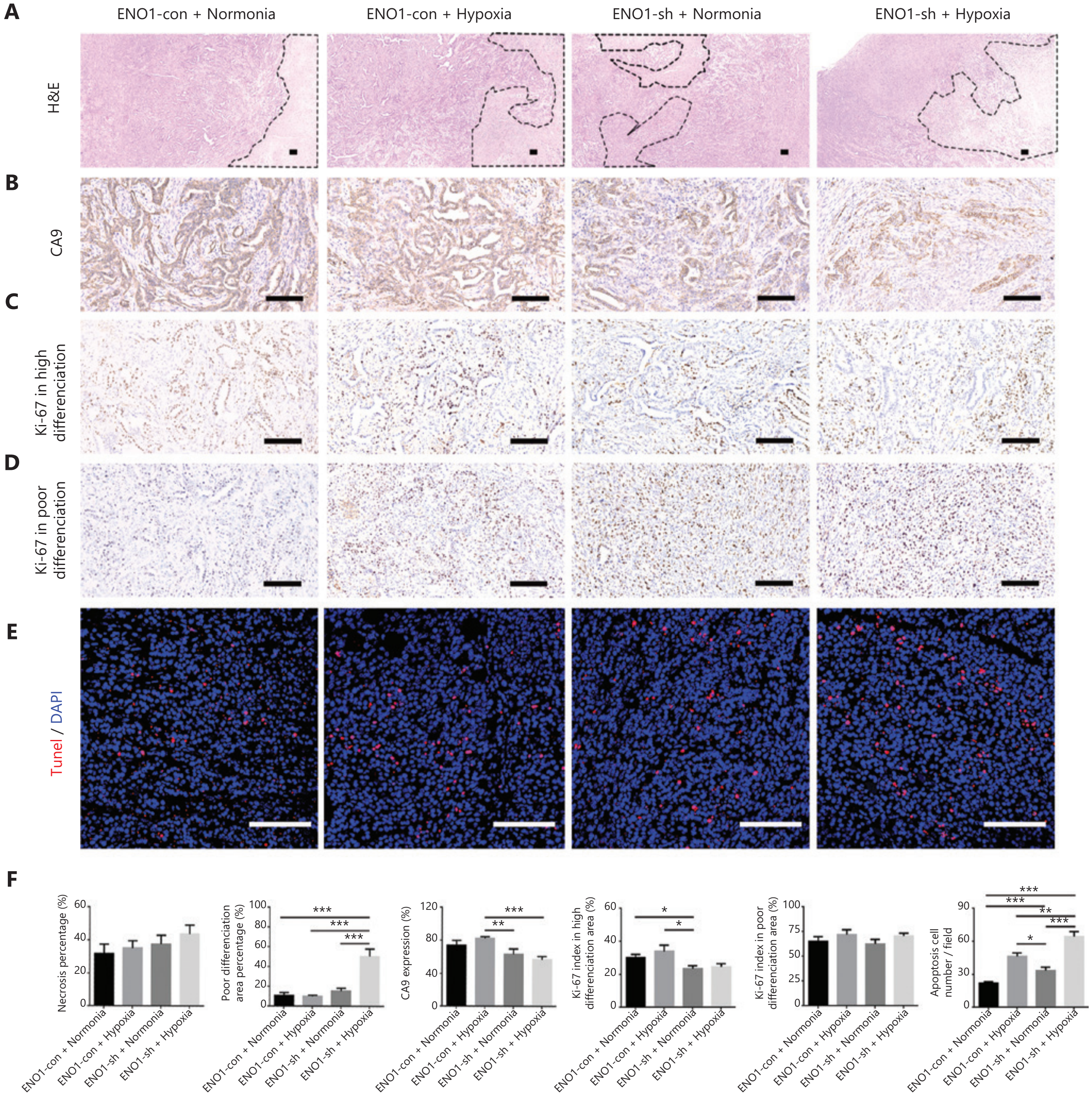

Figure S6 Comparison of tumor proliferation, hypoxic area, and cell death of SW1990-shENO1 and SW1990-con pancreatic cancer cells in ischemic and control hindlimbs. (A) Hematoxylin-eosin staining of SW1990-shENO1 and SW1990-con pancreatic cancer cells in ischemic and control hindlimbs. Dashed lines encircle the necrotic area. (B) CA9 expression of SW1990-shENO1 and SW1990-con pancreatic cancer cells in ischemic and control hindlimbs. (C) Ki-67 expression in the high differentiation area of SW1990-shENO1 and SW1990-con pancreatic cancer cells in ischemic and control hindlimbs. (D) Ki-67 expression in the poor differentiation area of SW1990-shENO1 and SW1990-con pancreatic cancer cells in ischemic and control hindlimbs. (E) Apoptotic SW1990-shENO1 and SW1990-con pancreatic cancer cells in ischemic and control hindlimbs. Red: TUNEL; blue: DAPI. (F) Quantification of necrosis, CA9 expression, Ki-67 index, and apoptosis of SW1990-shENO1 and SW1990-con pancreatic cancer cells in ischemic and control hindlimbs. Scale bars = 100 μm. *$P < 0.05$, **$P < 0.01$, ***$P < 0.005$.

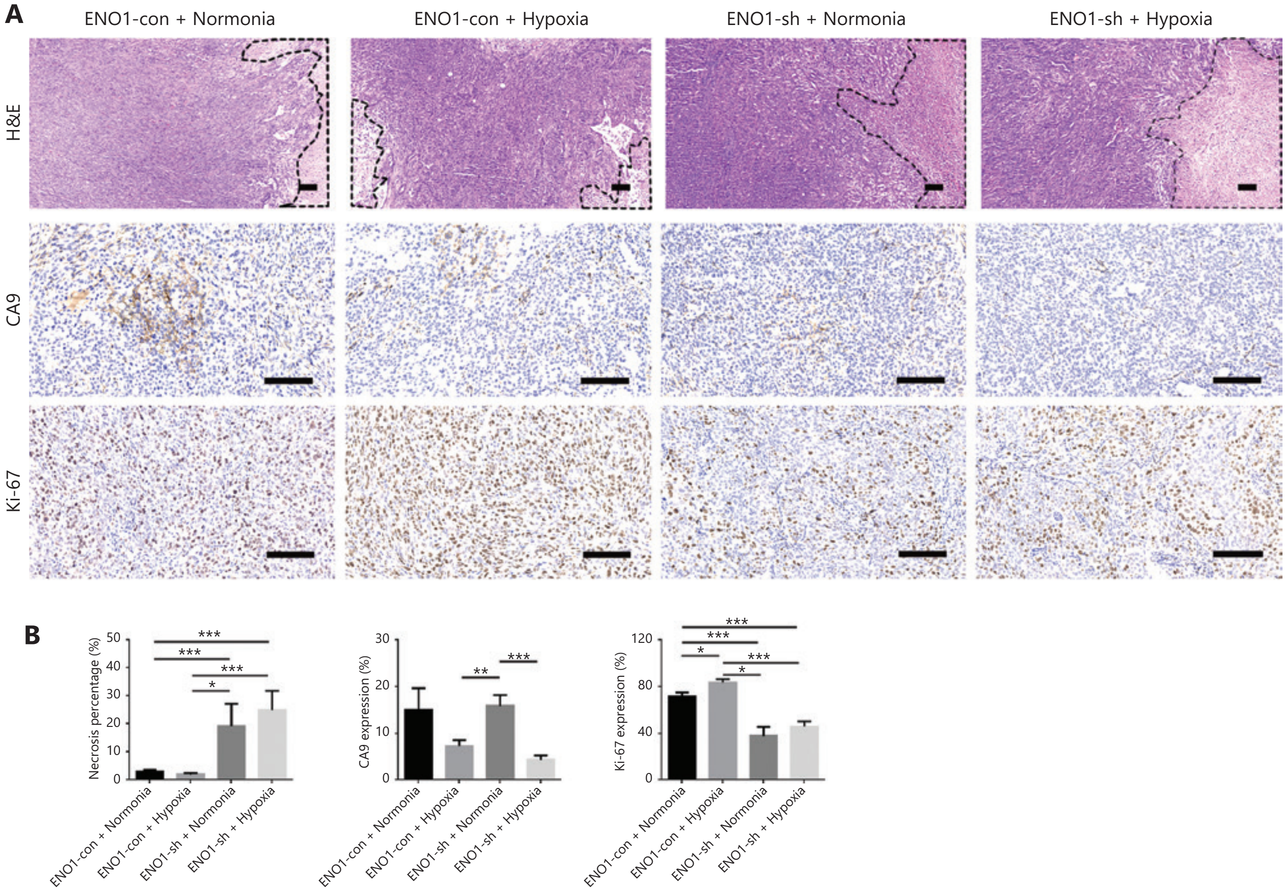

Figure S7 Comparison of tumor proliferation and hypoxic area of PANC-1-shENO1 and PANC-1-con pancreatic cancer cells in ischemic and control hindlimbs. (A) Hematoxylin-eosin staining of PANC-1-shENO1 and PANC-1-con pancreatic cancer cells in ischemic and control hindlimbs. Dashed lines encircle the necrotic area. CA9 expression of PANC-1-shENO1 and PANC-1-con pancreatic cancer cells in ischemic and control hindlimbs. Ki-67 expression of PANC-1-shENO1 and PANC-1-con pancreatic cancer cells in ischemic and control hindlimbs. (B) Quantification of necrosis, CA9 expression, and Ki-67 index of PANC-1-shENO1 and PANC-1-con pancreatic cancer cells in ischemic and control hindlimbs. Scale bars = 100 μm. *$P < 0.05$, **$P < 0.01$, ***$P < 0.005$.

Table S1 Comparison of pathological and clinical features between high ENO1 expression and low ENO1 expression in 84 pancreatic cancer cases.

Parameter		Low ENO1 expression, *n*	High ENO1 expression, *n*	χ^2	*P*
Gender	Male	37(74.0%)	17(50.0%)	5.077	0.022
	Female	13(26.0%)	17(50.0%)		
Age	< 50	3 (6.0%)	6 (17.6%)	2.807	0.092
	≥ 50	50 (94.0%)	34 (82.4%)		
Tumor volume	0 < T < 2	5 (10.0%)	1 (2.9%)	5.739	0.043
	2 ≤ T < 4	30 (60.0%)	17 (50%)		
	4 ≤ T	15 (30.0%)	16 (47.1%)		
Differentiation	High to moderate	25 (50.0%)	22 (64.7%)	3.552	0.042
	Low	25 (50.0%)	12 (35.3%)		
Invasion of pancreatic capsule	No	14 (28.0%)	9 (26.5%)	0.048	0.485
	Yes	36 (72.0%)	25 (73.5%)		
Lymph node metastasis	No	43 (86.0%)	9 (82.4%)	0.412	0.333
	Yes	7 (14.0%)	25 (17.6%)		
Intravascular tumor thrombus	No	46 (92.0%)	31 (87.5%)	0.036	0.531
	Yes	4 (8.0%)	3 (8.8%)		
Nerve invasion	No	40 (80.0%)	24 (70.6%)	1.976	0.111
	Yes	10 (20.0%)	10 (29.4%)		
Vascular invasion	No	44 (88.0%)	27 (79.4%)	2.282	0.099
	Yes	6 (12.0%)	7 (20.6%)		

Table S2 Correlation between ENO1 expression and pathological and clinical features in 84 pancreatic cancer cases.

Parameter	*r*	*P*
Gender	0.285	0.000***
Age	−0.202	0.009**
Tumor volume	0.003	0.966
Differentiation	−0.033	0.672
Invasion of pancreatic capsule	0.031	0.686
Lymph node metastasis	−0.025	0.749
Nerve invasion	0.180	0.020*
Vascular invasion	0.095	0.221
CA19-9 level in serum	0.167	0.036*
CA242 level in serum	0.084	0.446
CEA level in serum	0.134	0.220
CA9 expression	0.126	0.039*

$^*P < 0.05$, $^{**}P < 0.01$, $^{***}P < 0.001$.
